# Point-of-care Ultrasound Diagnosis of Tennis Leg

**DOI:** 10.5811/cpcem.2018.11.41022

**Published:** 2019-01-07

**Authors:** Aaron J. Monseau, Brenden J. Balcik, Nicolas Denne, Melinda J. Sharon, Joseph J. Minardi

**Affiliations:** * West Virginia University School of Medicine, Department of Emergency Medicine, Morgantown, West Virginia; † West Virginia University School of Medicine, Department of Medical Education, Morgantown, West Virginia

## Abstract

A 38-year-old male presented with left calf pain after a fall while skiing. Physical examination revealed tenderness over the gastrocnemius with a palpable mass and pain with resisted plantar flexion. Point-of care-ultrasound (POCUS) of the gastrocnemius was consistent with a muscle rupture, and we made a diagnosis of tennis leg. The patient was instructed to rest for two weeks, followed by a home rehabilitation program, and he was able to return to his normal activities. Here we present a case of tennis leg quickly and accurately diagnosed with POCUS, negating the need for additional advanced imaging.

## INTRODUCTION

Patients frequently present to the emergency department (ED) with musculoskeletal injuries. The majority of the workup in the ED has long centered on evaluation for fracture with plain radiographs. Once significant fractures have been effectively ruled out, most physicians will send these patients to follow-up with their primary care physician, a physician of sports medicine, or an orthopedist. Tendon ruptures have been evaluated with ultrasound in the ED when clinically appropriate, which should speed up diagnosis and thus improve patient outcomes. Muscular injuries such as full tears, partial tears, or even large contusions can be well diagnosed with point-of-care ultrasound (POCUS), which should ensure correct and prompt treatment. We present one such case of a muscular tear.

## CASE REPORT

A 38-year-old white male who is an avid skier presented with left calf pain described as sharp on the medial aspect that started after he fell while skiing about two weeks prior. During the fall, his left ankle was forced into extreme dorsiflexion. He had immediate onset of severe pain and had to stop skiing that day. He noticed swelling in the medial calf along with bruising of the area over the next few days. After about a week, he was able to ski again but had pain aggravated by active plantar flexion.

On physical examination of his left leg, his knee and ankle both appeared normal and had normal strength and range of motion with no tenderness. Sensation and pulses were normal. There was tenderness over the medial head of his gastrocnemius with a palpable, firm, four-centimeter ovoid mass. Pain was elicited during resisted plantar flexion. His right leg was completely normal.

**Image 1:**
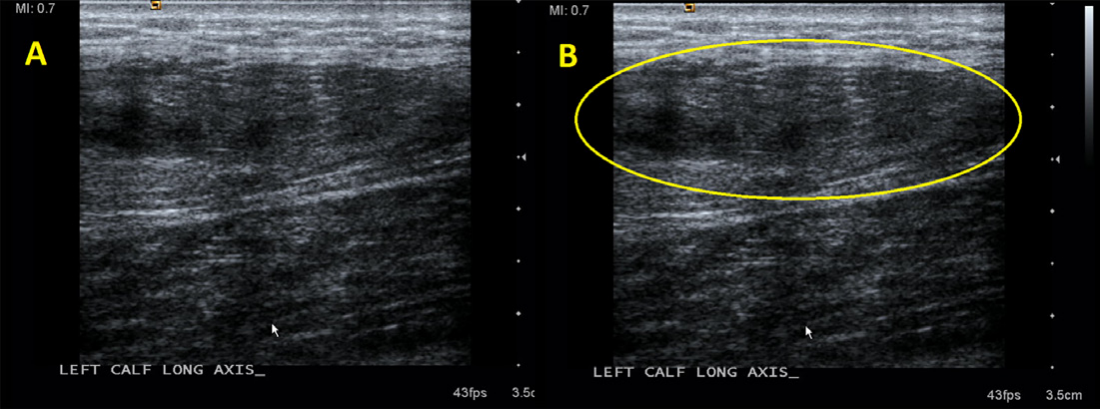
In frame A, the enlarged, disorganized, heterogeneous tissue of a muscle tear is seen in long axis. These findings are circled in frame B.

**Image 2:**
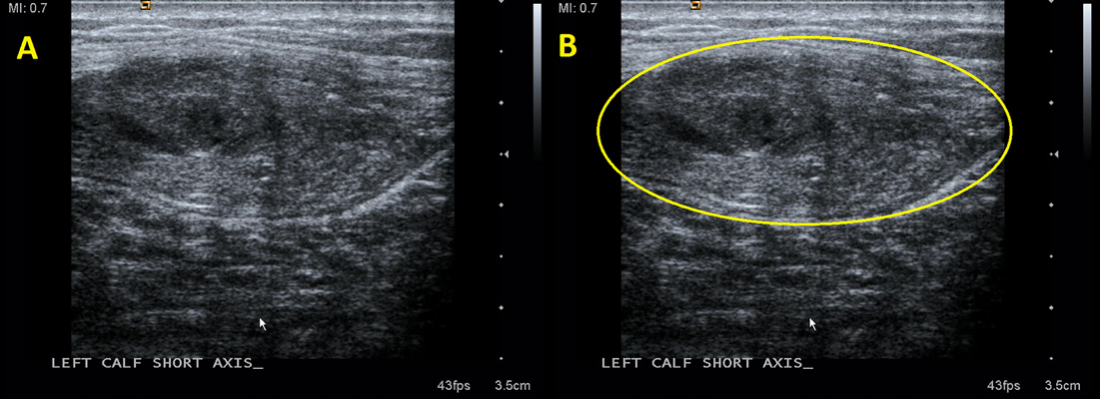
In frame A, the enlarged, disorganized, heterogeneous tissue of a muscle tear is seen in short axis. These findings are circled in frame B.

**Image 3:**
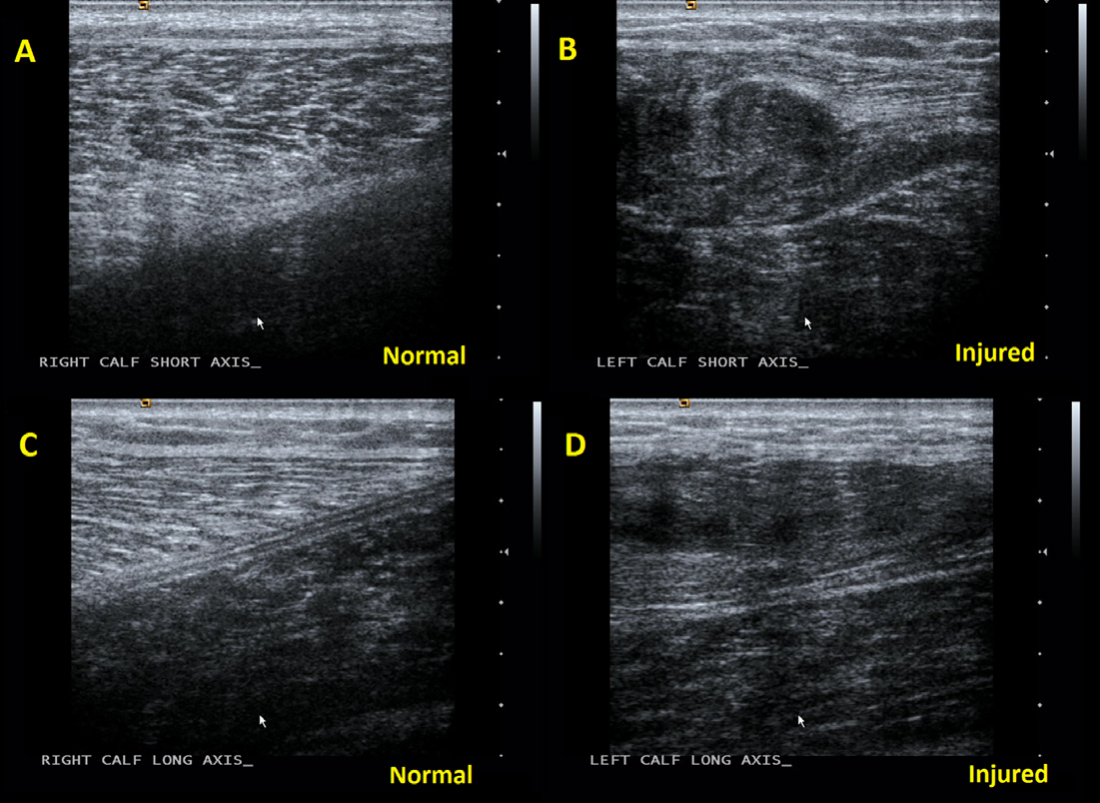
Frame A is a comparison view of the unaffected gastrocnemius muscle in the same patient. Here the organized regular appearance of alternating dark muscle and bright connective tissue characteristic of normal muscle belly in short axis is appreciated, in comparison to the injured gastrocnemius muscle in frame B where the regular pattern is disrupted by the swollen, heterogeneous, and disorganized appearance of a muscle tear. Frame C shows the normal appearing, linearly arranged alternating dark muscle and bright connective tissue in the unaffected gastrocnemius in long axis. Frame D shows the disrupted, irregular, and heterogeneous appearance of muscle tear in the injured gastrocnemius.

Radiographs of the lower leg obtained in the ED were normal. A POCUS was then performed in the ED with specific focus over the mass in the medial head of the gastrocnemius, revealing a swollen, heterogeneous, disorganized mass in the medial head of the gastrocnemius, as demonstrated in Images 1–3. Comparison views to the unaffected extremity, as seen in Image 3, further clarified the findings. We made a diagnosis of muscle tear in the medial head of the gastrocnemius.

The patient was instructed on exercises for strengthening and range of motion of the calf and ankle and specifically eccentric exercises that he should complete after an initial rest period of two weeks. On follow-up two months after the initial visit, his symptoms had nearly completely resolved. He reported mountain biking several times per week with only minimal pain after a long ride. The mass in the calf resolved, and he relayed no concerns about his leg for the upcoming ski season.

## DISCUSSION

Tennis leg, which previously indicated a plantaris tendon rupture, is now more commonly a term used to describe rupture of the medial head of the gastrocnemius muscle.^1^ Tennis leg is a common injury seen in middle-aged athletes with a male predominance.^2^ Mechanism of injury typically involves knee extension with concomitant forced dorsiflexion of the ankle or active plantar flexion of the foot during simultaneous knee extension.^3^,^4^ These mechanisms are commonly encountered in patients playing tennis, running, or jumping, but may occur with more routine activities such as walking up stairs, and has even been reported to occur during Namaz prayer.^1^,^2^,^5^ Patients will typically hear an audible “pop” and feel a pulling or tearing sensation within the calf at the time of injury.^1^,^6^ Clinical findings include calf swelling and tenderness, and pain with weight bearing.^4^ Patients will frequently hold the foot in plantar flexion, which avoids placing tension on the gastrocnemius.^6^

With the initial history and physical, the differential diagnosis would include muscle strain, muscle tear, Achilles tear, Achilles tendinosis, symptomatic Baker’s cyst, and deep venous thrombosis. While magnetic resonance imaging can be used to evaluate gastrocnemius muscle injury, high cost in addition to availability issues, limit its utility.^4^ We propose the use of POCUS to aid with the diagnosis of tennis leg and other acute musculoskeletal disorders. Point-of-care ultrasonography is a rapid, repeatable, noninvasive, and low-cost method of evaluating muscle injury, which can also be used to evaluate the full differential diagnosis with a high degree of accuracy in these cases.^4^ Data on the use of ultrasound in the diagnosis of tennis leg comes largely from case literature and reports by imaging specialists. There are few reports documenting the use of ultrasound at the point of care by clinicians to make the diagnosis.

**CPC-EM Capsule**

What do we already know about this clinical entity?

*Tears of the medial head of the gastrocnemius can be common, especially in active individuals. Previously, magnetic resonance imaging (MRI) was frequently used for diagnosis*.

What makes this presentation of disease reportable?

*With point-of-care ultrasound (POCUS) for diagnosis, the patient can avoid a long stay in the emergency department and the higher cost associated with MRI*.

What is the major learning point?

*POCUS for muscle tears is a relatively simple skill to learn, especially for emergency physicians who are already adept at using POCUS*.

How might this improve emergency medicine practice?

*Using POCUS instead of MRI can significantly decrease both length of stay for patients and overall healthcare costs*.

Muscle ultrasonography is typically accomplished with high-frequency linear array transducers (7.5–13 MegaHertz). The most important sonographic finding is interruption of the normally uniform, homogeneous, linear appearance of the muscle, which can typically be identified at the patient’s point of maximal pain. Typically, an enlarged, heterogeneous, and disorganized mass is seen at the area of muscle injury. Other findings may include hematomas and fluid collections, which are more common with more severe tears and correspond with prolonged recovery time. Comparison views to the contralateral extremity are useful.^7^ The muscle lesions become more hypoechoic later in the course, decreasing in size with eventual formation of scar tissue at six-month to one-year follow-up.^3^,^7^

Ultrasound has proven to be very useful to make the diagnosis, provide prognostic information, and evaluate other differential considerations.^7^ Bianchi et al. retrospectively reviewed sonographic images of 65 patients with clinically suspected tennis leg and described the typical sonographic features and sonographic progression of the diagnosis.^4^ Flecca et al. prospectively performed ultrasound examinations of 35 consecutive patients with clinical features of tennis leg and described the findings. Interestingly, other diagnoses such as phlebitis and a ruptured Baker’s cyst were discovered in six patients.^2^

Treatment for a rupture of the medial head of the gastrocnemius is typically non-operative with surgery reserved for refractory cases.^8^ In the acute period, treatment revolves around rest, ice, compression and elevation. Crutches may be needed due to pain. Once the patient is able to bear weight a rehabilitation protocol can be developed where the patient gradually increases resistance against movement of the ankle progressing from resistance bands to calf-raises with body weight or more. As with many muscle tears, the patient may see a significant improvement in the first few weeks, but most will not feel back to normal for six to eight weeks or more. The available literature suggests that the severity of findings on ultrasound may correlate with the course.^3^

## CONCLUSION

While a few studies have investigated the usefulness of ultrasound by imaging specialists, POCUS is an ideal modality for the diagnosis of a tear of the medial head of the gastrocnemius, also known as “tennis leg,” due to its bedside availability, low cost, accuracy, and utility in investigating alternate diagnoses. In addition, making the diagnosis at the bedside may decrease the need for subsequent imaging and clinic visits related to injury. Finally, during the literature review for this article, we found no other cases where a tear of a medial head of the gastrocnemius resulted from skiing as the ski boot likely protects against this injury.

## References

[1] Pachecho RA, Stock H (2013). Tennis leg: mechanism of injury and radiographic presentation. Conn Med.

[2] Flecca D, Tomei A, Ravazzolo N (2007). US evaluation and diagnosis of rupture of the medial head of the gastrocnemius (tennis leg). J Ultrasound.

[3] Shah JR, Shah BR, Shah AB (2010). Pictorial essay: Ultrasonography in ‘tennis leg’. Indian J Radiol Imaging.

[4] Bianchi S, Martinoli C, Abdelwahab IF (1998). Sonographic evaluation of tears of the gastrocnemius medial head (“tennis leg”). J Ultrasound Med.

[5] Yilmaz C, Orgenc Y, Ergenc R (2008). Rupture of the medial gastrocnemius muscle during namaz praying: An unusual cause of tennis leg. Comput Med Imaging Graph.

[6] Sarwark JF (2010). Essentials of Musculoskeletal Care.

[7] Kwak HS, Han YM, Lee SY (2006). Diagnosis and follow-up US evaluation of ruptures of the medial head of the gastrocnemius (“tennis leg”). Korean J Radiol.

[8] Cheng Y, Yang HL, Sun ZY (2012). Surgical treatment of gastrocnemius muscle ruptures. Orthop Surg.

